# Multicellular Tumour Spheroid as a model for evaluation of [^18^F]FDG as biomarker for breast cancer treatment monitoring

**DOI:** 10.1186/1475-2867-6-6

**Published:** 2006-03-23

**Authors:** Azita Monazzam, Pasha Razifar, Martin Simonsson, Fredrik Qvarnström, Raymond Josephsson, Carl Blomqvist, Bengt Långström, Mats Bergström

**Affiliations:** 1 Department of Oncology, Radiology and Clinical Immunology, Uppsala University, SE-751 85 Uppsala, Sweden; 2 Uppsala University, Centre for Image Analysis, Lägerhyddsvägen 3, SE-752 37 Uppsala, Sweden; 3 Department of Pharmaceutical Biosciences, Uppsala University, Sweden; 4 Uppsala Imanet AB (PET Center), BOX 967, Sweden; 5 Novartis Pharma AG, Clinical Imaging, CH-4002 Basel, Switzerland; 6 Department of medical Science, The Academic Hospital, S-751 85 Uppsala, Sweden

## Abstract

**Background:**

In order to explore a pre-clinical method to evaluate if [^18^F]FDG is valid for monitoring early response, we investigated the uptake of FDG in Multicellular tumour spheroids (MTS) without and with treatment with five routinely used chemotherapy agents in breast cancer.

**Methods:**

The response to each anticancer treatment was evaluated by measurement of the [^18^F]FDG uptake and viable volume of the MTSs after 2 and 3 days of treatment.

**Results:**

The effect of Paclitaxel and Docetaxel on [^18^F]FDG uptake per viable volume was more evident in BT474 (up to 55% decrease) than in MCF-7 (up to 25% decrease).

Doxorubicin reduced the [^18^F]FDG uptake per viable volume more noticeable in MCF-7 (25%) than in BT474 MTSs.

Tamoxifen reduced the [^18^F]FDG uptake per viable volume only in MCF-7 at the highest dose of 1 μM.

No effect of Imatinib was observed.

**Conclusion:**

MTS was shown to be appropriate to investigate the potential of FDG-PET for early breast cancer treatment monitoring; the treatment effect can be observed before any tumour size changes occur.

The combination of PET radiotracers and image analysis in MTS provides a good model to evaluate the relationship between tumour volume and the uptake of metabolic tracer before and after chemotherapy. This feature could be used for screening and selecting PET-tracers for early assessment of treatment response.

In addition, this new method gives a possibility to assess quickly, and in vitro, a good preclinical profile of existing and newly developed anti-cancer drugs.

## Background

Positron emission tomography (PET) is a multi-purpose non-invasive imaging technique with a wide range of applications both in vivo and in vitro [1]. In clinical oncology PET has been used for diagnosis, staging, and restaging after treatment or recurrence of different malignancies, including breast cancer [2-4].

PET with 2-fluorine-18-fluoro-2-deoxy-D-glucose ([^18^F]FDG-PET) represents a functional imaging modality that is based on metabolic characteristics of malignant tumours [5]. The uptake of [^18^F]FDG into tissue reflects both transport and phosphorylation of glucose by viable cells. Quantitative assessment of tumour metabolism by [^18^F]FDG-PET represents a novel approach in screening the response of malignant tumours to chemotherapy [6-12].

Early assessment of response would greatly benefit management of patients receiving chemotherapy by assuring continuance of effective therapy in those who respond or instituting alternative therapy in those who do not [11]. It would also be beneficial if patients with unresponsive tumours could be identified at a much earlier stage, thereby avoiding the use of ineffective, toxic and expensive treatment. As an innovative instrument for therapy monitoring, PET provides a more timely assessment of the efficacy of specific therapies, which would then offer a significant alteration in clinical management.

However, PET with a tracer for cellular function might not necessarily allow an early assessment of treatment response. A prerequisite for this is that the treatment affects biochemical cascades leading to antitumoral actions, and that the action record by the PET tracer is in some way mechanistically coupled to these cascades. E.g. inhibition of growth by alkylation of DNA might not necessarily lead to inhibition of [^18^F]FDG uptake and phosphorylation. Therefore it is not advisable to start a clinical trial with an antitumoral agent expecting PET to be used for treatment monitoring, without an initial assessment that PET can indeed serve this task.

MTSs represent a transitional level between cells growing as an in vitro monolayer and solid tumours in experimental animals or patients [13-16]. It has been confirmed that the cytology and the morphology of spheroids resembles that of experimental tumours in mice and natural tumours in humans before neovascularisation. Hence the MTS model has gained fundamental importance in therapeutically oriented investigations, as in the areas of radiotherapy, chemotherapy, photodynamic therapy, radioimunotherapy, and cell- and antibody-based immunotherapy [17-19].

Here MTS model was used to investigate the capability of [^18^F]FDG-PET to monitor early response of five commercially available and routinely used chemotherapy agents. Microscope-image-analysis of MTS was implemented to relate cell viability to [^18^F]FDG-uptake. We found the combination of morphological imaging and assessment of metabolic condition of tumour improved the quantitative knowledge about tumour and treatment response.

## Materials and methods

### Cell lines

1. Cells of the MCF-7 human breast cancer line (European Collection of Cell Culture) were grown in MEM/EBSS supplemented with 10% FCS, 1 mM sodium pyruvate, 2 mM L-glutamine, 1% non-essential amino acid and 5% penicillin (Tamro). The medium was changed twice weekly and cells were maintained in exponential growth phase.

2. Cells of the BT474 human breast cancer line (American Type Culture Collection) were grown in DMEM-high glucose supplemented with 10% FCS, 1 mM sodium pyruvate, 2 mM L-glutamine and 5% penicillin (Tamro). The medium was changed twice weekly and cells were maintained in exponential growth phase.

### Multicellular tumour spheroid

The tumour cells were trypsinized from the stem monolayer culture. Cell suspensions were then seeded in 24-well, 1% agarose-coated spheroid plates, with approximately 50,000 cells per well for MCF-7 cell line and 10,000 cells per well for BT474 cell line. The spheroids were kept at 37°C with 5% CO_2_, and grown for five days.

### Image analysis (SASDM)

Images of MTSs were recorded and analyzed in semi-automated size determination software (SASDM)[20]. Total, necrosis and viable volume of each MTS was calculated by the program.

### ^18^F-labeled 2-Fluoro-D-glucose ([^18^F]FDG) uptake

The MTSs were incubated for 50 min at 37°C with 0.5 ml medium per well containing 3MBq [^18^F]FDG[21], and then washed 3 × 5 min with medium (1 ml/well). Finally MTSs with 20 μl washing medium were transferred to 5 ml tubes and [^18^F]FDG uptake was evaluated in a calibrated well γ-counter. A 20 μl sample of the incubation medium was measured as reference, and 20 μl from the last wash medium was measured as background control.

The [^18^F]FDG-uptake in aggregates was defined as:

Total uptake = (Act conc. (Bq/ml) of aggregate) / (Act conc. (Bq/ml) of reference)

### Basic [^18^F]FDG experiments

To ensure the physiological validity of [^18^F]FDG uptake in MTS, a number of basic experiments were performed:

• *Temperature dependence of [^*18*^F]FDG uptake*. The [^18^F]FDG uptake experiments were performed with two different incubation temperatures: 4°C and 37°C.

• *Competition Experiment*. Glucose concentration in the growing culture medium was 5 mM. To perform the competition experiment, unlabeled extra glucose was diluted in the growing culture medium with a concentration of 0 mM (for the control group), 5 mM, 10 mM and 20 mM. Culture medium was replaced by glucose/ [^18^F]FDG-containing culture medium. After 50 min incubation the [^18^F]FDG uptake was measured.

• *Inhibition Experiment*. The MTSs were pre-incubated with four different concentrations of an inhibitor of glucose transport, Cytochalasin-B; 0 μM (for control group), 10 μM, 25 μM and 50 μM followed by 50 min [^18^F]FDG incubation.

• *Insulin-mediated [^*18*^F]FDG uptake*. The MTSs were pre-incubated with or without (control group) 10 μM insulin followed by 50 min [^18^F]FDG incubation.

It is noticeable that the basic experiments were performed only on MTSs of MCF-7 cell line. In each experimental set-up six MTSs in one 24-well plate were referred to as one group and the experiments were repeated three times.

### Anticancer treatment

The anticancer agents used were:

• Paclitaxel (an inhibitor of microtubule remodelling)

• Docetaxel (also an inhibitor of microtubule remodelling)

• Doxorubicin (an anthracycline antibiotic)

• Tamoxifen (an anti-oestrogen)

• Imatinib (a protein-tyrosine kinase inhibitor).

Each drug was diluted in the growing culture medium to a concentration of 10 nM, 100 nM and 1 μM. Treatment begun at day five of MTS growth with change of the culture medium to the drug-containing medium. After 1 h the medium was renewed, and the MTSs remained in drug containing medium for 2–3 days.

For the MCF-7 cell line, each group included 6 replicates and the experiments were repeated twice.

For the BT474 cell line, each group included 4 replicates and the experiments were repeated three times.

The response to each anticancer treatment was evaluated by measurement of the [^18^F]FDG uptake and viable volume of the MTSs after 2 and 3 days of treatment.

### Immunohistochemistry

Paraffin embedded MTSs were cut in 4 μm sections. Immunohistochemical staining were performed in a Benchmark IHC/ISH (Ventana Medical Systems, Tucson, Arizona) using a monoclonal antibody against γ-H2AX, dilution 1:50, (Upstate, Charlottesville, Virginia). Staining were enzymatically developed with 3, 3-diaminobenzidine tetra hydrochloride (DAB) and counterstained with hematoxylin.

### Image analysis (γ-H2AX)

Images of MTS were acquired using a Spot Insight Colour CCD camera (Diagnostic Instruments, Sterling Heights, Michigan) coupled to a Nikon Eclipse E400, with a Nikon 10x objectives (Nikon Corporation, Tokyo, Japan) and saved as 24-bit RGB TIFF.

The area of γ-H2AX positive cells in the viable part of the MTS was determined using semi-automated image analysis and compared to the total viable area. The image analysis software was written in Java™ as a plug-in to ImageJ. The viable region of the MTS was selected manually and the γ-H2AX positive cells were selected using a fixed threshold in the blue channel.

### Analysis of data

[^18^F]FDG uptake per viable volume of each MTS and γ-H2AX positive area of each slice was normalized to the control group. All data were analysed by ANOVA and Dunnett's Multiple Comparison Test in Graph Pad Prism.

## Results

### Basic [^18^F]FDG experiments

The tumour cells formed multicellular spheroids about 1 day after seeding. Within the next day the characteristic rounded shape with a rim of viable cells and a central necrosis was observed.

**Figure 1:**
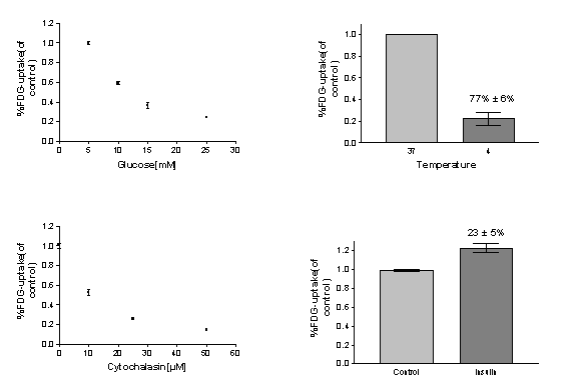
Competition experiment (upper left) Temperature dependence of [^18^F]FDG -uptake (upper right) Inhibition experiment (lower left) Insulin dependence glucose transport (lower right).

When an increasing concentration of glucose was added to the incubation medium, [^18^F]FDG uptake in MTSs gradually decreased (Fig 1). The concentration inducing a 50% reduction compared to control was 10 mM. A reduced temperature during the [^18^F]FDG incubation reduced the [^18^F]FDG uptake by 77% (SE = 6%) (Fig 1). These two observations indicate a saturable and temperature-dependent process for the [^18^F]FDG uptake and hence exclude dominance by passive diffusion.

When the glucose-transport inhibitor Cytochalasin B was added to the incubation medium, [^18^F]FDG uptake significantly decreased; e.g., a 50% inhibition occurred at 12 μM (Fig 1). Addition of insulin to the incubation medium increased the [^18^F]FDG uptake by 23% (SE = 5%) (Fig 1). These two observations suggest glucose transport is the dominant mechanism for [^18^F]FDG uptake in the MTSs.

### [^18^F]FDG uptake alteration

• Paclitaxel and Docetaxel

Taxanes significantly reduced the uptake of [^18^F]FDG. The decrease in uptake per viable volume was more evident in BT474 (up to 55% decrease) than MCF-7 (up to 25% reduction).

• Doxorubicin

Doxorubicin, which damages DNA by intercalation, reduced [^18^F]FDG uptake per viable volume at the highest dose, more noticeable in MCF-7 (25%) than in BT474 MTSs.

• Tamoxifen

Tamoxifen, an anti-oestrogen compound, reduced [^18^F]FDG uptake per viable volume in MCF-7 at the highest dose of 1 μM.

• Imatinib

Imatinib, a tyrosine kinase inhibitor, slightly increased [18F]FDG uptake in MCF-7 at the intermediate dose of 100 nM and 2 days follow up, otherwise no effect was observed.

**Figure 2:**
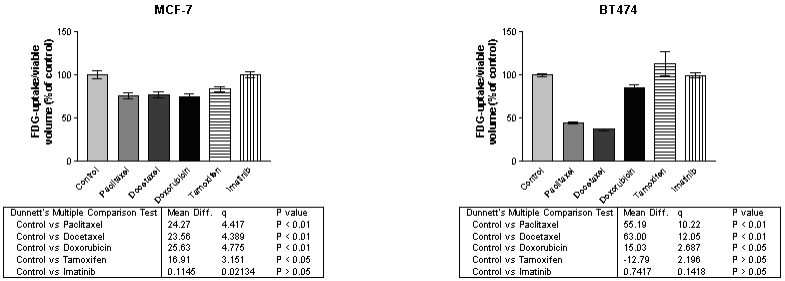
Effect of 3 days treatments with 1μM chemotherapy agents observed in FDG-uptake in MTS of MCF-7 and BT474.

Figure 2 illustrates [^18^F]FDG uptake alteration in MTSs.

### Immunohistochemistry

**Figure 3:**
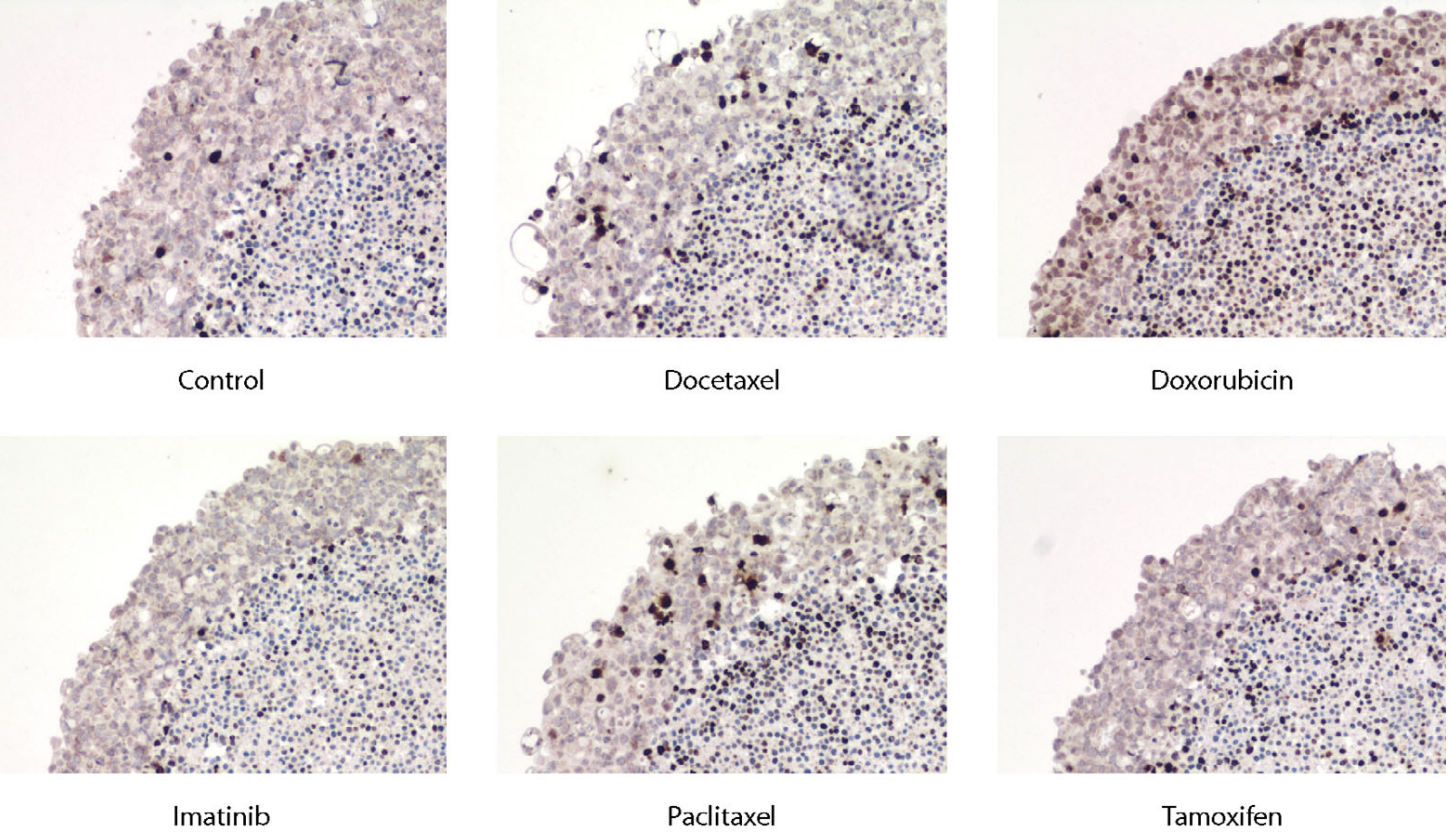
γH2AX staining in MTS of MCF-7 after 3 days.

**Figure 4:**
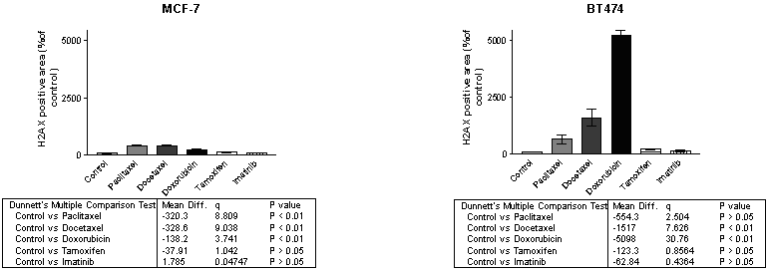
Effect of 3 days treatments with 1 μM chemotherapy agents, observed in γH2AX staining in MTS of MCF-7 and BT474

γH2AX formation in MCF-7 (Fig. 3) was considerably increased in Taxanes- and Doxorubicin treated MTSs. In BT-474 the increase was more noticeable in Doxorubicin treated MTSs (Fig. 4).

**Figure 5:**
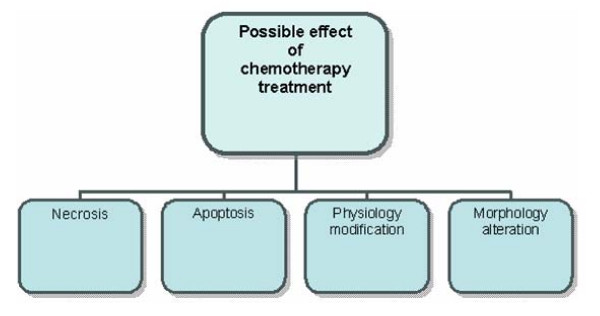
Schematic of possible effect of chemotherapy treatment.

## Discussion

In order to explore if a range of conventional anti-cancer drugs would impact on the cellular uptake of FDG, we utilized multicellular tumour spheroids, treated with the drugs and after a few days of treatment evaluated the uptake of FDG. Two different breast cancer cell lines were selected with different characteristics. BT474 cells have up-regulated mRNA and HER2/neu tyrosine kinase-linked receptor protein in comparison to MCF7, while the expression of the estrogen receptor alpha is known to be up-regulated in MCF7 cells. These two cell-lines are assumed to be representative for a variety of breast cancers.

Although growing cells as MTS is more laborious than monolayer, MTS cells more closely mimic the real biological environment by providing cell-to-cell adhesion and signalling. This gives a more relevant picture of the drug effects by including limitations in penetration, distribution and feedback mechanisms in cell signalling.

Here we established a new methodology for potential drug screening by combining measurement of the morphologic drug effect and evaluation of PET tracer uptake. The method could serve dual purposes: to evaluate effects of new drugs and to identify the optimal PET biomarker for early evaluation of drug effect.

Initially we investigated the nature of [^18^F]FDG uptake in MTS. We verified a reduced metabolism after either challenging with low temperature, or competition by unlabeled glucose or inhibition with Cytochalasin-B. We also observed a transport increase with insulin addition. These sets of tests confirmed that in MCF-7 MTSs, [^18^F]FDG accumulation is a biomarker which is related to transport via the specific glucose transporters GLUT, followed by phosphorylation by hexokinase.

An important aspect in the evaluation of chemotherapy by PET-FDG is whether [^18^F]FDG can be used as a biomarker to indicate treatment response. The accepted criterion for treatment response is reduction in tumour size or verified lack of further growth. This can in turn relate to a variety of functional aspects, such as induction of apoptosis, direct cell kill, effects on vascularity, reduced proliferation, remodelling of proportions of tumour and other cells etc. These aspects do not necessarily relate to effects on FDG uptake. E.g. transient increase in uptake of FDG has been postulated to be an indication of a positive effect causing a temporary inflammatory response, metabolic flare. A retardation of cellular proliferation can be associated with a lack of effect on FDG uptake. Hence it is of utmost importance to clarify the relation between antitumoral effects and effects on FDG uptake. For this, we believe that MTS are the best in vitro cellular model, allowing an easy evaluation of FDG uptake plus a means to observe effects on cellular growth. The method is ideally associated with evaluations of apoptosis induction, proliferation and other cellular biomarkers.

Paclitaxel and Docetaxel has a known activity against a broad range of tumour types, including breast, ovarian, lung, head and neck cancers [22-24]. These potent anti-neoplastic drug binds to the N-terminal region of b-tubulin and promotes the formation of highly stable microtubules that resist depolymerization, thus preventing normal cell division and arresting the cell cycle at the G2-M phase [23-26]. Combining [^18^F]FDG-PET and image analysis, we monitored the effect of Paclitaxel for 3 days on two different human breast cancer line, in a concentration of 10 nM, 100 nM and 1 μM, added after 5 days of growth as MTS. In BT474, we observed reduced glucose transport per viable volume already at the lowest dose and the shorter treatment time. The effect was more moderate in MCF-7. These results indicate disturbance in cells physiology as a consequence of the treatment with taxanes that can be recorded with [^18^F]FDG-PET.

Doxorubicin is an anthracycline antibiotic produced by the fungus streptomyces peucetius. It damages DNA by intercalation of the anthracycline portion, metal ion chelation, or by generation of free radicals. Doxorubicin has also been shown to inhibit DNA topoisomerase II which is critical to DNA function [24]. Cytotoxic activity is not specific to cell cycle phase [27]. Doxorubicin is also known to be activated by mitochondrial electron transport system and causes mitochondrial energy failure. Thus, the effect might be not only by DNA damage, but also by energy metabolism damage[28]. In our experiments Doxorubicin decreased glucose transport at the highest dose. The data suggest that [^18^F]FDG would record an antitumoral effect of Doxorubicin in the early phase of treatment at relevant doses. The effect of Doxorubicin was more intense on MCF-7 than on BT474. γH2AX formation that indicates DNA fragmentation [29] was drastically increased in Doxorubicin treated BT474 MTSs. This radically alteration was not observed in FDG-uptake. It opens the discussion if [^18^F]FDG is always the ideal biomarker for follow-up of all category of anticancer treatment.

Tamoxifen is a synthetic non-steroidal anti-oestrogen. It is thought to competitively block oestrogen receptors. Other biochemical effects of Tamoxifen include interaction with protein kinase C and stimulation of human NK cells [23,24,30]. As expected, the effect of Tamoxifen can only be observed in MCF-7. In our experiments the effect of Tamoxifen can only be related to a glucose-transport alteration [31].

Imatinib mesylate is a protein-tyrosine kinase inhibitor that inhibits the Bcr-Abl tyrosine kinase, the constitutive abnormal tyrosine kinase created by the Philadelphia chromosome abnormality in chronic myeloid leukemia (CML)[32]. It inhibits proliferation and induces apoptosis in Bcr-Abl positive cell lines as well as fresh leukemic cells from Philadelphia chromosome positive chronic myeloid leukemia [33].

Imatinib is not entirely selective; it also inhibits the receptor tyrosine kinases for platelet-derived growth factor (PDGF), stem cell factor (SCF), c-Kit and thereby inhibits PDGF- and SCF-mediated cellular events [34]. In our experiments no effect of Imatinib was observed.

## Conclusion

To conclude, the combination of PET radiotracers, image analysis of growth pattern and necrosis induction plus histochemical analyses of proliferation and apoptosis in MTSs provides a good model to evaluate the relationship between tumour volume and the uptake of metabolic tracer before and after chemotherapy. This feature could be used for screening and selecting PET biomarkers for early assessment of treatment response.

In addition, this new method gives a possibility to assess quickly, and in vitro, a good preclinical profile of existing and newly developed anti-cancer drugs.

Used clinically on biopsies, this method could potentially be used on an individual level, first to select a treatment for a particular patient, and then to select the PET tracer for monitoring in a short follow up time the optimal drug and dose regimen.

## Competing interests

The author(s) declare that they have no competing interests.

## Authors' contributions

Authors AM, PR and MB helped with the design of the study. They created the method for applying SASDM, performed the image and data analysis and drafted the manuscript.

MS and FQ developed and performed the image and data analysis of the γ-H2AX staining.

Authors RJ, CB and BL helped with some of the practical approaches and the writing of the paper.

The authors wish to express their gratitude to Mrs. Veronika Asplund-Eriksson and Mrs. Maj-Lis Book for their contributions to this study.
